# Reducing power requirements for high-accuracy decoding in iBCIs

**DOI:** 10.1088/1741-2552/ad88a4

**Published:** 2024-11-01

**Authors:** Brianna M Karpowicz, Bareesh Bhaduri, Samuel R Nason-Tomaszewski, Brandon G Jacques, Yahia H Ali, Robert D Flint, Payton H Bechefsky, Leigh R Hochberg, Nicholas AuYong, Marc W Slutzky, Chethan Pandarinath

**Affiliations:** 1 Wallace H. Coulter Department of Biomedical Engineering, Emory University and Georgia Institute of Technology, Atlanta, GA, United States of America; 2 Department of Neurology, Northwestern University, Chicago, IL, United States of America; 3 Center for Neurotechnology and Neurorecovery, Department of Neurology, Massachusetts General Hospital, Harvard Medical School, Boston, MA, United States of America; 4 Veterans Affairs Rehabilitation Research & Development Center for Neurorestoration and Neurotechnology, Providence VA Medical Center, Providence, RI, United States of America; 5 Robert J. & Nancy D. Carney Institute for Brain Science and School of Engineering, Brown University, Providence, RI, United States of America; 6 Department of Neurosurgery, Emory University, Atlanta, GA, United States of America; 7 Department of Cell Biology, Emory University, Atlanta, GA, United States of America; 8 Department of Neuroscience, Northwestern University, Chicago, IL, United States of America; 9 Department of Biomedical Engineering, Northwestern University, Evanston, IL, United States of America; 10 Department of Physical Medicine and Rehabilitation, Northwestern University, Chicago, IL, United States of America; 11 Shirley Ryan AbilityLab, Chicago, IL, United States of America

**Keywords:** brain-computer interfaces, neural dynamics, low power, neural decoding

## Abstract

*Objective.* Current intracortical brain-computer interfaces (iBCIs) rely predominantly on threshold crossings (‘spikes’) for decoding neural activity into a control signal for an external device. Spiking data can yield high accuracy online control during complex behaviors; however, its dependence on high-sampling-rate data collection can pose challenges. An alternative signal for iBCI decoding is the local field potential (LFP), a continuous-valued signal that can be acquired simultaneously with spiking activity. However, LFPs are seldom used alone for online iBCI control as their decoding performance has yet to achieve parity with spikes. *Approach.* Here, we present a strategy to improve the performance of LFP-based decoders by first training a neural dynamics model to use LFPs to reconstruct the firing rates underlying spiking data, and then decoding from the estimated rates. We test these models on previously-collected macaque data during center-out and random-target reaching tasks as well as data collected from a human iBCI participant during attempted speech. *Main results.* In all cases, training models from LFPs enables firing rate reconstruction with accuracy comparable to spiking-based dynamics models. In addition, LFP-based dynamics models enable decoding performance exceeding that of LFPs alone and approaching that of spiking-based models. In all applications except speech, LFP-based dynamics models also facilitate decoding accuracy exceeding that of direct decoding from spikes. *Significance.* Because LFP-based dynamics models operate on lower bandwidth and with lower sampling rate than spiking models, our findings indicate that iBCI devices can be designed to operate with lower power requirements than devices dependent on recorded spiking activity, without sacrificing high-accuracy decoding.

## 1. Introduction

Intracortical brain-computer interfaces (iBCIs) can restore functional capabilities for people with paralysis by monitoring cortical neural activity and mapping it to an external variable [1, 2], such as intended cursor movements, actuations of a robotic effector, handwritten characters, spoken words, and even muscle contractions [3–19]. These devices typically use implanted electrodes to measure spiking activity, which in this work refers to unsorted threshold crossing events consisting primarily of action potentials. Spikes are the predominant signal used to train iBCI decoding algorithms to translate neural activity into control signals for external effectors [1]. Recent advances in neural interfaces have raised the prospect of implantable wireless iBCI devices [20–22], which promise to offer further benefits to users by making devices untethered, safer, and more portable.

A key consideration in designing wireless iBCIs is the power required for data collection and transmission, as high power requirements necessitate larger batteries to ensure sufficient battery life to last between recharges or replacements. While advantageous for decoding performance, spikes are at a disadvantage in terms of power consumption. To reliably identify spikes, voltages are acquired at high sampling rates (e.g. 30 kHz). While spiking activity can be binned and digitized to lower power requirements for wireless data transmission, the high sampling rate and bandwidth necessitate power-hungry amplifiers and analog-to-digital converters [23]. Recent studies have demonstrated that spikes can be extracted from lower bandwidth signals [20, 24], but the bandwidth must remain sufficiently high to avoid inaccurate firing rate estimates or degradations in decoding performance [23].

Reducing the necessary signal bandwidth can allow more flexibility in engineering options, as devices that use less power and require smaller implanted batteries raise fewer safety concerns with regards to the heating of biological tissues and lessen the demand on users imposed by frequent battery replacement surgeries or recharging. Continuous features such as spike band power (SBP; 300–1000 Hz) offer a promising avenue for lowering front-end power consumption of iBCI devices by restricting the bandwidth of the signal of interest, relative to spiking activity [23]. However, decreasing the signal and recording bandwidths further necessitates looking at even lower frequency bands, some of which have been shown to have strong relationships to both spikes and motor behavior [25–33]. Of interest in this study were signals containing frequencies less than or equal to 450 Hz, which we refer to as local field potentials (LFPs) due to their predominantly low frequency content (despite some overlap with the spike band). LFPs have successfully been used to decode behavior in iBCIs, both on their own and to supplement spikes [3, 5, 27, 28, 34–36]. In addition, though here we focus on decoding within a single day, LFPs are often cited for having greater longevity throughout recording implant lifetime and improved decoding stability over spikes [34, 36–38]. Yet, when spikes are readily available, LFPs are not often used alone in iBCIs, as no studies to date have shown that LFPs can achieve performance on par with spikes-based decoders.

Neural population dynamics modeling provides a potential avenue for achieving high-performing LFP-based iBCI decoders. Dynamics models aim to uncover temporal patterns underlying the activity of populations of neurons [39–47]. In doing so, models typically yield a low-dimensional latent representation of the neural activity (‘factors’) and denoised neural firing rates. These model outputs often have a close correspondence to behavior, which can lead to improved behavioral decoding performance—a priority for next generation iBCIs [40, 42, 45].

While both LFPs and spikes have been previously modeled as dynamical systems [29, 48, 49], we propose a novel paradigm for LFP-based dynamics models where LFP power is used as input, and the model’s objective is to reconstruct the firing rates underlying spiking activity. In this paradigm, training the model requires LFPs and spikes to be collected simultaneously. However, after training, only LFPs are required to perform model inference and obtain firing rate estimations on new timepoints. Thus after an initial model training dataset is collected, further use of the model relies only on the LFP, a low-power signal.

Here we demonstrate that LFP-based dynamics models, as a pre-decoding step for iBCIs, can lower power requirements while maintaining the decoding performance of high-power, spikes-based counterparts. We begin by calculating the power requirements of various wireless iBCI circuit components for each signal modality, and find that recording LFPs in place of spikes can reduce power consumption by an order of magnitude or more. We then demonstrate model performance on three datasets. First, we show that for a variety of frequency bands and data resolutions, LFP-based dynamics models trained on a monkey center-out dataset yield accurately-reconstructed firing rates and high-performance decoding comparable to that of spikes-based dynamics models. Next, we show that on a less structured monkey random target reaching task, LFP-based dynamics models maintain their ability to accurately reconstruct firing rates and decode behavior. Finally, we investigate a human iBCI speech task and find that LFP-based dynamics models performed comparably to spikes-based dynamics models for phoneme decoding.

In all, we demonstrate that LFP-based dynamics models produce outputs that can be used to train decoders performs better than LFP power alone and comparably to spikes-based dynamics models. This decoding advantage is accompanied by a decrease in required power consumption to collect model input data post-training. Lower power consumption benefits the development of wireless iBCIs by enabling longer battery life or the transmission of more channels. Further, given the previously-demonstrated longevity of LFP signals, improving the decoding performance achievable with LFPs alone helps extend the functional lifetime of iBCI implants as spiking data quality degrades. Overall, these results demonstrate that models of neural population dynamics can substantially improve the potential of LFPs to be used in real-world iBCIs.

## 2. Methods and materials

### 2.1. Nonhuman primate data collection

We analyzed previously-collected primate data from two reaching tasks [28, 36, 50]. A rhesus macaque was implanted with a 96-channel microelectrode array (Blackrock Neurotech, Inc.) in the arm area of primary motor cortex (M1). The monkey performed each reaching task with the arm contralateral to the array. We collected broadband data from each electrode at 30 kHz using a 128-channel acquisition system (Cerebus, Blackrock Neurotech, Inc.). To extract spikes, we high pass filtered the broadband data (1st order causal filter, 300 Hz cutoff) and thresholded it using a threshold manually set for each channel (average threshold = 5.2 standard deviations above mean waveform potential). To extract LFPs, we first bandpass filtered the broadband data from 0.3 to 500 Hz (1st order causal filter), then resampled the signal at 2 kHz, and finally notch filtered it at harmonics of 60 Hz for powerline noise removal.

The first task we analyzed was an eight-target center-out reaching task. On each trial, the monkey began by holding at the center of a 10 cm-radius circle of targets for 0.5–0.6 s. Then, one of eight 2 cm square targets spaced at 45° intervals around the circle was illuminated. The monkey had to reach the outer target within 1.5 s and hold for a random time between 0.2–0.4 s to obtain a liquid reward. The second task was a random target reaching task. On each trial, the monkey had to acquire a series of 6 randomly positioned targets appearing one-at-a-time, holding each for 0.1 s, to obtain the reward. The targets spanned the majority of the 20-by-20 cm workspace.

### 2.2. Human subject data collection

Participant T16 is a participant in the BrainGate2 clinical trial (ClinicalTrials.gov Identifier: NCT00912041). This pilot clinical trial was approved under an investigational device exemption (IDE) by the US Food and Drug Administration (IDE #G090003; CAUTION: Investigational device. Limited by federal law to investigational use.). Permission was also granted by the Mass General Brigham IRB (protocol #2009P000505) and the Emory University IRB (protocol #00003070). T16 is a right-handed woman, 52 years of age at the time of the study, with tetraplegia and dysarthria due to a pontine stroke that occurred approximately 19 years prior to study enrollment. We placed four 64-channel intracortical microelectrode arrays (Blackrock Microsystems, Salt Lake City, UT; 1.5 mm electrode length) in her left precentral gyrus. In this study, we analyze data collected on trial day 69 from only one of these arrays, which was located in the speech-related ventral precentral gyrus (6v). We recorded and processed broadband data at 30 kHz using the Backend for Realtime Asynchronous Neural Decoding (BRAND) platform [51]. To extract the spiking data, we first re-referenced the data using linear regression referencing (LRR) [52] with respect to data collected immediately before the period of interest. We then bandpass filtered from 250 to 5000 Hz (4th order Butterworth filter) and finally identified threshold crossings with a threshold of −4.5RMS. We extracted LFPs by processing the broadband data to be consistent with the nonhuman primate data as described above: we first low pass filtered the data with a 1000 Hz cutoff (5th order Butterworth filter), downsampled the signal to a 2 kHz sampling rate, and notch filtered at harmonics of 60 Hz to remove powerline noise.

We asked participant T16 to perform a cued speech task in which she vocalized a word presented to her on a screen [53], similar to previous speech-related tasks [17, 19]. The word bank was compiled from the 50 word vocabulary introduced by Moses *et al* [14]. At the beginning of each trial, a red square appeared on the screen directly below a single word. After a delay period of 1500 ms, the square turned green, cueing the participant to vocalize the word to the best of her ability. After it was clear the participant was done speaking, an experimenter ended the trial. There was a 1000 ms interval before the next trial began. All research sessions were performed at the participant’s place of residence.

### 2.3. Data preprocessing

#### 2.3.1. LFPs

Before analysis, we further preprocessed raw LFPs by identifying and removing disconnected or overly active channels, computing LFP power, and causally normalizing the resulting signals. For some analyses, we also applied a Gaussian kernel to smooth the signal (standard deviation = 30 ms for monkey data, 50 ms for T16 data).

To compute LFP power, we first took the raw 2 kHz LFP signals and computed a short-time Fourier transform (STFT) using a frequency resolution of 5 Hz (except for the 0–8 Hz band, for which we used 2 Hz) and shifting the window by 10% of the window length at each step (for the 0–8 Hz band, 4%). We then identified the magnitudes in the frequency bands of interest and computed power by summing their values squared into 20 ms bins. For determining channels to remove, we took these power values and computed the mean within each channel. We excluded channels that had mean power in the frequency band of interest that was less than 50% of the median of the per-channel means (assumed disconnected) [54] or greater than or equal to twice the 99th quantile of the per-channel means (overly active). For modeling and other decoding-based analyses, we then computed the log of these power values. We causally normalized the power by z-scoring it in each timestep using means and standard deviations computed from a 3 min rolling window.

#### 2.3.2. Spikes

For the random target dataset, we first removed coincident spikes by zeroing the value at any time step at which a spike occurred on more than 30% of the channels. For all datasets, we also removed any channels involved in correlations with other channels higher than 0.2 when computed in 1 ms bins prior to modeling; this prevents model overfitting to correlated noise events across channels. Then, we resampled spikes into 20 ms bins by computing the cumulative sum of spike counts in each 20 ms time window. For some analyses, we also smoothed the spikes by convolving with a Gaussian kernel (standard deviation = 30 ms for monkey data, 50 ms for T16 data).

#### 2.3.3. Behavior

We analyzed behavior and performed neural decoding by looking at windows around a movement alignment time. For both monkey reaching datasets, we computed an alignment time as the time at which the speed in the window starting 250 ms after trial start time crosses the threshold of 70% of the peak speed in each trial. We extracted the window of data 250 ms before to 500 ms after this alignment point. To avoid analyzing trials that may have had corrective movements or multiple speed peaks, we rejected any trial for which the first crossing computed from the start of the trial and the last crossing computed from the end of the trial did not match.

For the T16 data, we computed the envelope of microphone data collected during the session by mean-centering the data, high-pass filtering with a cutoff of 65 Hz, rectifying the signal, low-pass filtering with a cutoff of 10 Hz, and then downsampling the resulting envelope to 50 Hz to match the resolution of the neural data. To determine speech onset points, we adapted a custom algorithm to be applied to this envelope. Looking at the region between the go cue and trial stop time, we identified peaks in the differentiated envelope (positive peaks) and its inverse (negative peaks) to identify increases and decreases in the signal. We selected the first positive peak as the speech onset and the last negative peak as the speech offset. In order to reduce sensitivity to outliers or noise, we set a minimum threshold for peak magnitude of 3.5 to ensure that peak detection only captured large-amplitude changes in the differentiated microphone envelope.

### 2.4. Neural dynamics modeling

The neural dynamics model used in this work is latent factor analysis via dynamical systems (LFADS) [39, 40]. In short, LFADS models temporal patterns underlying neural activity using a series of recurrent neural networks. The input to the model is a neural signal $s\left( t \right)$. The Generator RNN models the generic dynamical system as $\dot x\left( {t} \right) = f\left( {x\left( t \right),{ }u\left( t \right)} \right)$. A Controller RNN models inputs to the dynamical system $u\left( t \right)$. Encoder RNNs model the initial conditions $x\left( 0 \right)$ and $u\left( 0 \right)$. The objective of the model is to best reconstruct the rates $\hat s\left( t \right)$ underlying the neural signal using a Poisson negative log likelihood (NLL) loss computed based on $s\left( t \right)$.

In the original model (termed ‘Spikes LFADS’ in this paper), $s\left( t \right)$ is comprised of binned spiking data, and $\hat s\left( t \right)$ is an estimate of the denoised firing rates learned by computing the Poisson NLL between $\hat s\left( t \right)$ and $s\left( t \right)$. In our work, we replaced the input data $s\left( t \right)$ with LFP power $p\left( t \right)$ (supp. figure 1). The input to the model is now $p\left( t \right)$, but the model’s objective is still to estimate $\hat s\left( t \right)$ by computing the Poisson NLL between $\hat s\left( t \right)$ and $s\left( t \right)$.

All neural data is modeled with LFADS in an unsupervised manner with respect to trial structure. The continuous data is divided into segments [55] as follows: 1000 ms windows with 200 ms overlap for center-out reaching datasets and 1000 ms windows with 350 ms overlap for random target reaching datasets and speech datasets. We trained LFADS models with fixed architecture parameters (supp. table 1) chosen based on previous work modeling monkey movement datasets [56] and adjusted based on empirical model performance. We optimized the hyperparameters of the LFP LFADS models using grid searches for both monkey datasets in which we selected hyperparameters based on model firing rate reconstruction (negative log likelihood) and post-hoc behavioral decoding performance on the modeled datasets (supp. tables 2 and 3). For these grid searches, we selected hyperparameters jointly over all five tested sessions for the monkey center-out task and for only one session for the monkey random target task. Due to task and decoder complexity, we optimized hyperparameters using only AutoLFADS [41] for the speech dataset. We also optimized the hyperparameters of all Spikes LFADS models using AutoLFADS. In addition, to aid in reducing overfitting to correlations between channels in the binned spiking data, we trained Spikes LFADS models with a data augmentation that randomly moved spikes up to 2 bins forward or backward in a different configuration on each training step [56].

After training, models trained on monkey datasets used a causal inference procedure to obtain resulting LFADS factors and denoised firing rates [51, 56]. The models performed inference using a sliding window of observed data; at each time step, one new bin of input data was added to the window, and the remaining time steps consisted of previously observed data. This resulted in one new bin of model output. In addition, rather than sampling from the posterior distribution many times and averaging, we used the means of the posterior distributions. These modifications help to best simulate an online iBCI scenario in which minimal latency is desired. Models trained on the speech dataset used standard acausal inference and posterior sampling as the decoder requires a window of data to operate, making millisecond-scale latencies less of a concern.

### 2.5. Quantification of firing rate reconstruction

To quantify how well a model’s firing rates represented the empirical firing rates, we computed the peri-stimulus time histogram (PSTH) *R^2^* between the model’s inferred firing rates and the smoothed spiking data (30 ms Gaussian) [57]. This value is computed using the condition averages in the window 250 ms before to 500 ms after the computed movement alignment time. We then concatenate each condition average along the time axis to compute *R^2^* for each channel and report the uniform averaged *R^2^* over channels.

### 2.6. Power consumption analysis

We assumed the wireless iBCI recording device components that may differ in power consumption between LFP and spike data acquisition include analog amplifiers, analog-to-digital converters, feature extractors, and wireless transmitters.

We calculated the power of the amplifier using the noise efficiency factor (NEF) formula [58]:

\begin{equation*}{P_{{\text{amp}}}} = {V_{{\text{source}}}}{\left( {\frac{{{\text{NEF}}}}{{{V_{{\text{RMS}}}}}}} \right)^2}\frac{{\pi \cdot {U_{\text{T}}} \cdot 4kT \cdot {\text{BW}}}}{2}\end{equation*}

where the voltage source ${V_{{\text{source}}}}$ was 3.3 V, the NEF was 4.0, the *V*_RMS_ was 2 *μ*V, the thermal voltage *U*_T_ was 26.7 mV, the Boltzmann constant $k$ is 1.38 × 10^−23^, the temperature was 310 K, and BW was the signal bandwidth [23].

We calculated the power of the analog-to-digital converter (ADC) by solving the Schreier Figure of Merit (FoM_s_) formula [59]:

\begin{align*}{P_{{\text{ADC}}}} = \frac{{{\text{BW}}}}{{{{10}^{\frac{{{\text{FoMs-SNDR}}}}{{10}}}}}}\end{align*}

where the FoM_s_ was 185 dB [60], SNDR was 96 dB, and BW was the sampling bandwidth (half of the sampling rate) [23].

We did not estimate power consumption for feature extraction because it has previously been shown that their consumption is orders of magnitude lower than that of the analog front-end [23]. For the data transmitter, we closely matched the transmission rate of the current state-of-the-art wireless iBCI recording device, as transmission rate is the driving factor behind transmitter power consumption [20, 24]. By matching transmission rate, data transmission power is matched between our proposed LFP and spikes circuits, allowing us to focus on potential power savings from the analog front-end when recording LFPs in place of spikes.

To mimic the potential data compression rates for spikes counted in 20 ms bins, we quantized the LFP power signal within the 150–450 Hz band for each session by first min-max scaling each channel of data with respect to the entire session (approximately 15 min of data) so that it falls within the range of 0–2^b^, where b is the number of bits in the resolution of interest, and flooring the resulting float values to integers. This simulates LFP data collection and compressed transmission from a wireless iBCI recording device. The resulting signal was used in two analyses. First, to assess whether data compression rates affect how well LFP power can predict behavior, we smoothed the quantized LFP power (Gaussian w/ standard deviation 30 ms) and trained a decoder. Later, to determine whether data compression rates had an impact on modeling performance, we used the quantized LFP power (unsmoothed) to train a neural dynamics model, and trained a decoder on the output rates.

We further assessed model performance at different LFP frequency bands by simulating a lower bandwidth signal. Here we considered the frequency bands 150–450 Hz, 100–200 Hz, 50–100 Hz, 25–50 Hz, and 0–8 Hz. We downsampled the raw LFP signal to the Nyquist frequency of the upper bound of the frequency range of interest (except for the 0–8 Hz band, for which we downsampled to 50 Hz, the lowest sampling rate to maintain our spiking bin size of 20 ms). Then, we computed LFP power and used the resulting features to perform model inference and decoding.

### 2.7. Neural decoding

For both nonhuman primate motor datasets, we applied a Wiener filter decoder with 4 time bins of history and L2 regularization of the form:

\begin{equation*}W\, = \,{\left({X^T}X\, + \,{R^T}R\right)^{ - 1}}{X^T}y\end{equation*}

where *W* is a matrix of filter coefficients, *X* represents the predictor neural data with history and bias, and *y* represents the output behavioral data. *R* represents a diagonal matrix with the L2 regularization constant filling the diagonal. The bias term was not regularized and therefore the diagonal entry of *R* was set to zero. To determine the optimal L2 value, we swept over 20 possible values spaced on a log scale (center out: 100-1000, random walk: 0.1-1000). We computed decoder weights using 10-fold cross-validation and reported the resulting *R*^2^ on a held-out validation set. The neural data we used to predict behavior was either a smoothed empirical signal (LFPs or spikes) or the LFADS outputs (latent factors or rates). To compute decoding accuracy, we used variance-weighted *R*^2^, defined as:

\begin{equation*}{R^2}\left( {y,\,\hat y} \right) = 1 - \frac{{{{\mathop \sum \nolimits}}_{d = 1}^D\,{{\mathop \sum \nolimits}}_{i = 1}^N{{\left( {{{\hat y}_{i,d}} - {y_{i,d}}} \right)}^2}}}{{{{\mathop \sum \nolimits}}_{d = 1}^D\,{{\mathop \sum \nolimits}}_{i = 1}^N{{\left( {{y_{i,d}} - {{\bar y}_d}} \right)}^2}}}.\end{equation*}

For the speech task, we used the recurrent neural network decoder described in Willett *et al* [19]. In brief, we passed preprocessed neural data through a linear layer, then into stacked gated recurrent units (GRU) RNNs, and finally into an output layer to produce phoneme predictions [17, 19]. We optimized model weights using connectionist temporal classification (CTC) loss, whose objective is to identify both when a new phoneme occurs and what the identity of that phoneme is. Each word was parsed into phonemes using the g2pE Python package [61], which provides labels in ARPAbet notation [62]. For each neural signal modality, we selected model architecture and hyperparameters by performing a random search of 200 decoder models. The neural data used to predict phonemes was either the smoothed raw signal (LFPs or spikes) or the LFADS output rates. We quantified decoding performance using the phoneme error rate (PER), defined as the edit distance of the decoded sequence (the number of substitutions, insertions, or deletions required to change the decoded sequence into the correct sequence) divided by the number of phonemes in the true sequence. In order to ensure model performance was consistent, we trained five decoders with different random seeds, using the same hyperparameters from the random search, and reported the mean and standard deviation across these models. To ensure that performance quantification focused on information within the neural signals themselves, we did not use a language model to perform error correction after phoneme decoding, in contrast to the original use case [19].

## 3. Results

### 3.1. LFPs require lower power consumption than spikes in a wireless iBCI circuit

**Figure 1.**
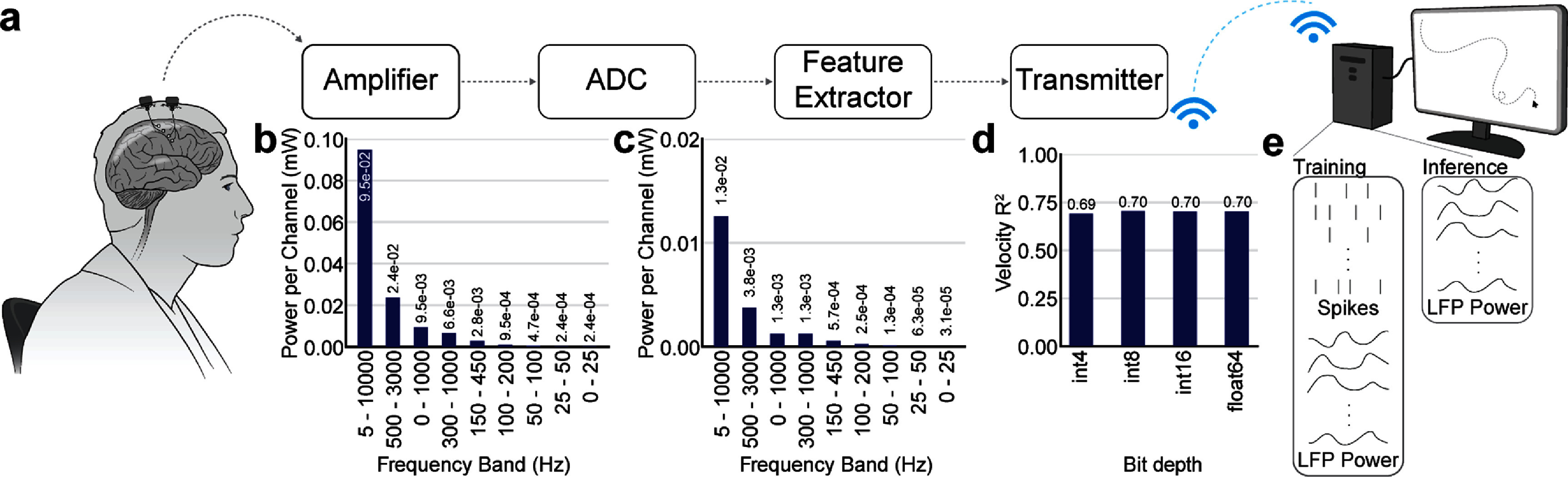
Schematic of wireless iBCI circuit and power advantages conferred by LFPs. (a) Schematic of circuit components in wireless iBCI. Neural activity is processed through an amplifier, analog-to-digital converter (ADC), feature extraction pipeline, and finally wirelessly transmitted to a computer where any further preprocessing, modeling, and decoding can take place. (b) Power consumed by the amplifier per channel of neural activity for different frequency bands. Those considered include high-bandwidth spikes (5–10000 Hz), low bandwidth spikes (500–3000 Hz), the raw LFPs used in this work (0–1000 Hz), SBP (300-1000 Hz), the band of LFP power used for dynamics modeling in this work (150–450 Hz), and lower LFP bands (100–200 Hz, 50-100 Hz, 25–50 Hz, 0–25 Hz). (c) Power consumed by the ADC per channel of neural activity for the same frequency bands shown in (b). (d) Decoding of LFP power in the 150–450 Hz band when represented at 4-bit, 8-bit, 16-bit, and 64-bit depth. (e) Components of neural activity required for training and performing inference with the proposed LFP-based dynamics model.

We first aimed to assess the theoretical magnitude of differences in power consumption between LFP- and spikes-based wireless iBCIs (figure 1(a)). Beginning with the amplifier, whose power requirements depend on the signal bandwidth, we computed the power consumed per channel of recorded neural data for a variety of frequency bands used to extract either spikes or LFPs (figure 1(b); see section 2). We estimated the frequency range of 5 Hz–10 kHz as a standard range for extracting spikes from high-bandwidth data; the amplifier in this range would consume 9.5 × 10^−2^ mW per channel of neural data. However, spikes can also be extracted with adequate accuracy from lower bandwidth signals, such as 500–3000 Hz [24]. In this frequency range, the amplifier would consume 2.4 × 10^−2^ mW per channel of neural data, only 25% of the power required for the high-bandwidth signal.

In this work, we largely analyzed LFP signals containing frequencies 0–1000 Hz, which offer further power benefits, requiring only 9.5 × 10^−3^ mW per channel. Recent work has also proposed SBP [23] as a low power signal, which would require 6.6 × 10^−3^ mW per channel. Better yet, our results largely used LFP power in the high-frequency band of 150–450 Hz; with further circuit optimizations, amplifier power consumption to collect a signal in this band would use only 2.8 × 10^−3^ mW of power per channel (12% of the required per-channel power of low-bandwidth spikes and 42% of the required per-channel power of SBP). Lower bandwidth LFP signals (100–200 Hz, 50–100 Hz, 25–50 Hz, 0–25 Hz), whose advantages may differ based on the decoding application, may reduce amplifier power consumption to as low as 2.4 × 10^−4^ mW per channel.

We next evaluated the power required by the analog-to-digital converter (ADC), which depends on the sampling bandwidth of the signal (figure 1(c)). Again, we found that high-bandwidth spikes would require the most power per channel (1.3 × 10^−2^ mW) followed by low-bandwidth spikes (3.8 × 10^−3^ mW). The LFP signal recorded at 2 kHz would provide a significant advantage, necessitating 1.3 × 10^−3^ mW. This value also holds for SBP as it requires collection at the same sampling rate and therefore has the same sampling bandwidth. Lower bandwidth signals recorded at their lower Nyquist sampling rates may even further lower ADC power requirements, with 150–450 Hz requiring only 5.7 × 10^−4^ mW (15% of that required for low-bandwidth spikes) and the lowest sampling bandwidth of 0–25 Hz requiring only 3.1 × 10^−5^ mW.

Moving through the circuit, LFPs would unquestionably reduce the amplifier and ADC power needed to acquire neural signals. The feature extraction step, which involves computing signal power or extracting threshold crossings and binning to the desired width, consumes a negligible amount of power relative to the analog front-end [23, 63]. We next investigated the transmission step, which wirelessly sends the neural data to an external computer for further processing and decoding.

A continuous-valued signal such as LFP, transmitted wirelessly at high precision, would require more transmission power than a discrete signal. To assess whether one could maintain high-performance decoding while transmitting low precision LFP signals, we analyzed previously-collected data from a monkey center-out reaching dataset. We first quantized the LFP power to different resolutions that might be used for data transmission (4-bit, 8-bit, 16-bit, and 64-bit; supp. figure 2). We then smoothed the resulting signal and predicted cursor velocity using a Wiener filter, and we found that decoding accuracy remained steady at all signal resolutions (figure 1(d)). As 4 bits per sample per channel was sufficient to preserve decoding, we estimated the transmission rate for 1024 channels for varying bin sizes and found it to range from 136.53 Kbps (30 ms bins) to 204.8 Kbps (20 ms bins). This is consistent with the current state-of-the-art wireless spikes-based iBCI, which operates at 163.84 Kbps to transmit spiking data from 1024 channels [20].

After wireless transmission of the neural data, further preprocessing as well as model and decoder training and inference steps would take place on an external machine with sufficient computational resources. Our proposed dynamics model uses LFP power to reconstruct spikes. Therefore, for initial model training, both LFP power and spikes would be required (figure 1(e)). This initial training dataset may be collected using a wired transmitter or with access to a high-power source such that power concerns are not at the forefront. After training, model inference only requires LFP power to yield estimates of firing rates that can be used for decoding. During this phase, we estimate significant power consumption advantages at the front-end using the amplifier and ADC and power consumption consistent with spikes-based devices for wireless transmission, saving at least 103.86 *μ*W per channel (96.8%) compared to high-bandwidth spikes, 24.05 *μ*W per channel (87.6%) compared to low-bandwidth spikes, and 4.48 *μ*W per channel (56.8%) compared to SBP.

### 3.2. LFP-based dynamics models accurately reconstruct spikes and enable power reduction

We modeled dynamics using latent factor analysis via dynamical systems (LFADS; see section 2.4). Briefly, LFADS approximates the dynamical system underlying a neural population using a series of recurrent neural networks (RNNs). In standard Spikes LFADS, the model input is observed spiking activity, and its objective is to minimize a lower bound on the likelihood of the observed spiking activity given the instantaneous firing rates it has estimated to underlie each channel of neural activity. We modified this scheme for LFP LFADS such that the model input is now the LFP power in one or more frequency bands, but the objective is still computed based on the likelihood of the spiking activity given estimated firing rates.

**Figure 2.**
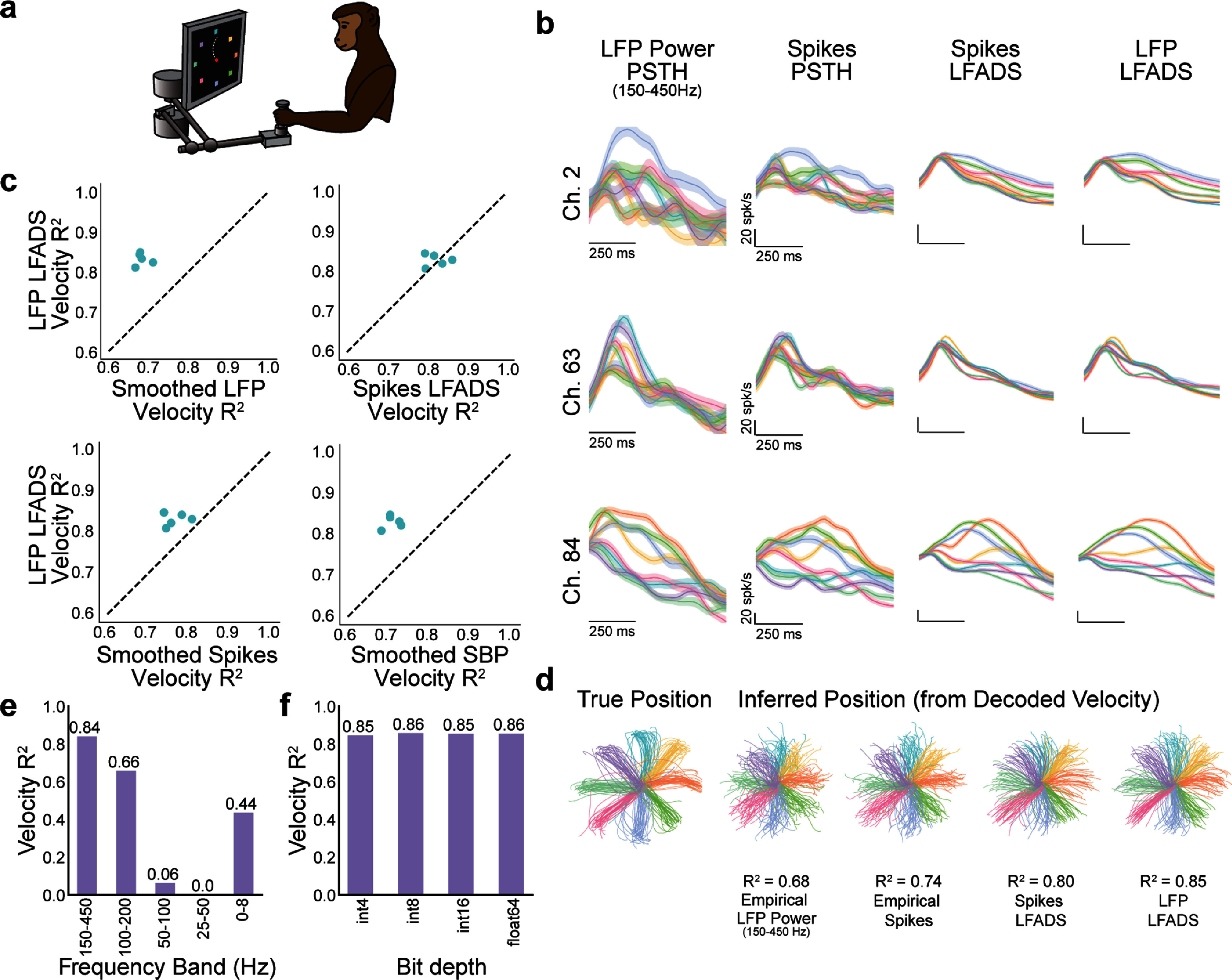
LFP-based dynamics models reconstruct firing rates and enable high-accuracy behavioral decoding in a monkey center-out reaching task. (a) Schematic of center-out reaching task. (b) Example PSTHs for three example channels from a single session for smoothed LFP power, smoothed spikes, Spikes LFADS rates, and LFP LFADS rates. Each color is a different reach direction, with solid lines indicating the trial average of neural activity for a given condition 250 ms before to 500 ms after the computed movement alignment time and shaded regions representing the standard error of the mean. (c) Velocity decoding performance (*R^2^*) for LFP LFADS compared to empirical LFP power (top left), Spikes LFADS (top right), smoothed spikes (bottom left), and smoothed SBP (bottom right). Each point represents the *R^2^* value for a model trained on one session of data; five sessions were evaluated in total. Dashed black line indicates unity. LFP power features from the 150–450 Hz band. (d) Left: Measured single trial reach trajectories, colored by target location for a single session. Right: reach position trajectories integrated from the decoded reach velocity when a decoder is trained on each of the four neural signal modalities. Data and *R*^2^ shown for the same single session as in (b). LFP power in the band 150–450 Hz, 64-bit resolution. (e) Decoding performance of LFP-based dynamics models when using input features from different frequency bands of LFPs to reconstruct spikes. (f) Decoding performance of LFP-based dynamics models when using LFP power features (150–450 Hz) computed at different resolutions.

We began by testing the performance of LFP-based dynamics models by applying them to the monkey center-out reaching task (figure 2(a)). We compared the denoised rates from the LFP LFADS model to those of a standard Spikes LFADS model and to the empirical firing rates estimated by smoothing the spikes with a Gaussian kernel. We found that despite the difference in input signal, LFP LFADS models reconstructed firing rates in a qualitatively similar manner to Spikes LFADS models (figure 2(b)), as evidenced by their peri-stimulus time histograms (PSTHs). Further, both models’ firing rate estimates were very similar to the empirical Spikes PSTHs, with PSTH *R^2^* values of 0.79 for Spikes LFADS and 0.85 for LFP LFADS.

To quantify the amount of behaviorally-relevant information captured by our models, we assessed the decoding performance from each neural signal modality using a Wiener filter trained to predict cursor velocity. We used five sessions of neural data recorded on different days. On each session, we trained an LFP LFADS model using LFP power in the 150–450 Hz band, and then trained a decoder from the LFADS rates to the cursor velocity. We compared the resulting decoding accuracies to those obtained by training decoders on various empirical neural signals, without any dynamics modeling. We first compared the decoding performance (*R*^2^) to that of training a decoder on the smoothed LFP Power from the same frequency band. We found that decoding from LFP LFADS rates (mean *R*^2^ = 0.83) exceeded the performance of decoding from empirical LFP Power (mean *R*^2^ = 0.68) for all sessions (*p*= 6.6 × 10^−5^ in one-sided t-test) (figure 2(c), top left). We further tested whether LFP LFADS decoding performance continued to exceed that of signals from a higher frequency band more often used for iBCI decoding, SBP (figure 2(c), bottom right). LFP LFADS exceeded the performance of decoding from smoothed SBP for all five tested datasets (mean *R*^2^ = 0.71, *p* = 1.26 × 10^−4^ in one-sided t-test), demonstrating that LFP LFADS can provide both power and decoding gains. Additionally, we ensured that LFP LFADS provided an advantage over traditional spikes decoding (figure 2(c), bottom left), where it again showed significantly higher performance over decoders trained on empirical smoothed spikes (mean *R*^2^ = 0.76, *p* = 4.01 × 10^−3^ in one-sided t-test).

Next, we wanted to compare the performance of decoders trained on LFP LFADS rates to those trained on Spikes LFADS rates. We trained separate Spikes LFADS models and decoders on each of the five sessions. We found that Spikes LFADS rates yielded decoding performance very comparable to the LFP LFADS counterpart (mean *R*^2^ = 0.82), with no significant difference between the groups (*p* = 0.64 in two-sided t-test) (figure 2(c), top right). These results were consistent with visualizations of the decoded cursor trajectories, shown for one session in figure 2(d). To ensure the quality of LFP LFADS model fits for an iBCI-like scenario, we also tested whether models could generalize to unseen neural data (supp. figure 3). Within the same recording day, velocity decoding performance and reconstruction of single-trial properties from LFP LFADS factors were comparable regardless of whether data was seen during model training.

Because the frequency band used for modeling thus far was relatively high, we wanted to further evaluate the performance of LFP-based dynamics models when trained on features from lower frequency bands (100–200 Hz, 50–100 Hz, 25–50 Hz, and 0–8 Hz; figure 2(e)). For each frequency band, we trained an LFP-based dynamics model on four sessions of data collected on the same calendar day and then trained a decoder on the output LFP LFADS rates. On a fifth session, we then downsampled the raw LFP signal to the Nyquist frequency of the upper bound of each frequency range prior to computing LFP power. Using this downsampled LFP power signal, we performed model inference with the trained LFP LFADS model to get the output LFADS rates, applied the trained decoder, and computed velocity *R^2^*. We found that while high-frequency LFP power (150–450 Hz) achieved the highest performance in terms of velocity decoding, some bands such as 100–200 Hz and 0–8 Hz offered reasonable decoding performance and may offer further benefits in amplifier or ADC power consumption. Other bands such as 50–100 Hz and 25–50 Hz yielded poor firing rate predictions with little relationship to behavior (near-zero decoding performance); this may be due to a lack of correspondence between the LFPs in these bands and the precise timing of spikes, which has been shown in previous work [25, 26].

Finally, we wanted to ensure that LFP-based dynamics models maintained their performance when using LFP features at a lower resolution (figure 2(f)). This is important to maintain the power required to transmit the data in our theoretical wireless iBCI recording device. We trained separate LFP-based dynamics models on an individual session of data (the same session used in figures 2(b) and (d)) after converting the LFP power features (150–450 Hz) to 4-bit, 8-bit, and 16-bit resolution. We found that no matter the resolution, the model yielded output rates that decoded velocity consistently, with negligible differences between them. Therefore, LFP-based dynamics models do not fail with lower resolution LFPs, allowing us to maintain the potential power savings of an iBCI recording device that were accrued using LFPs.

### 3.3. LFP-based dynamics models demonstrate high reconstruction and decoding performance in an unstructured monkey random target reaching task

**Figure 3.**
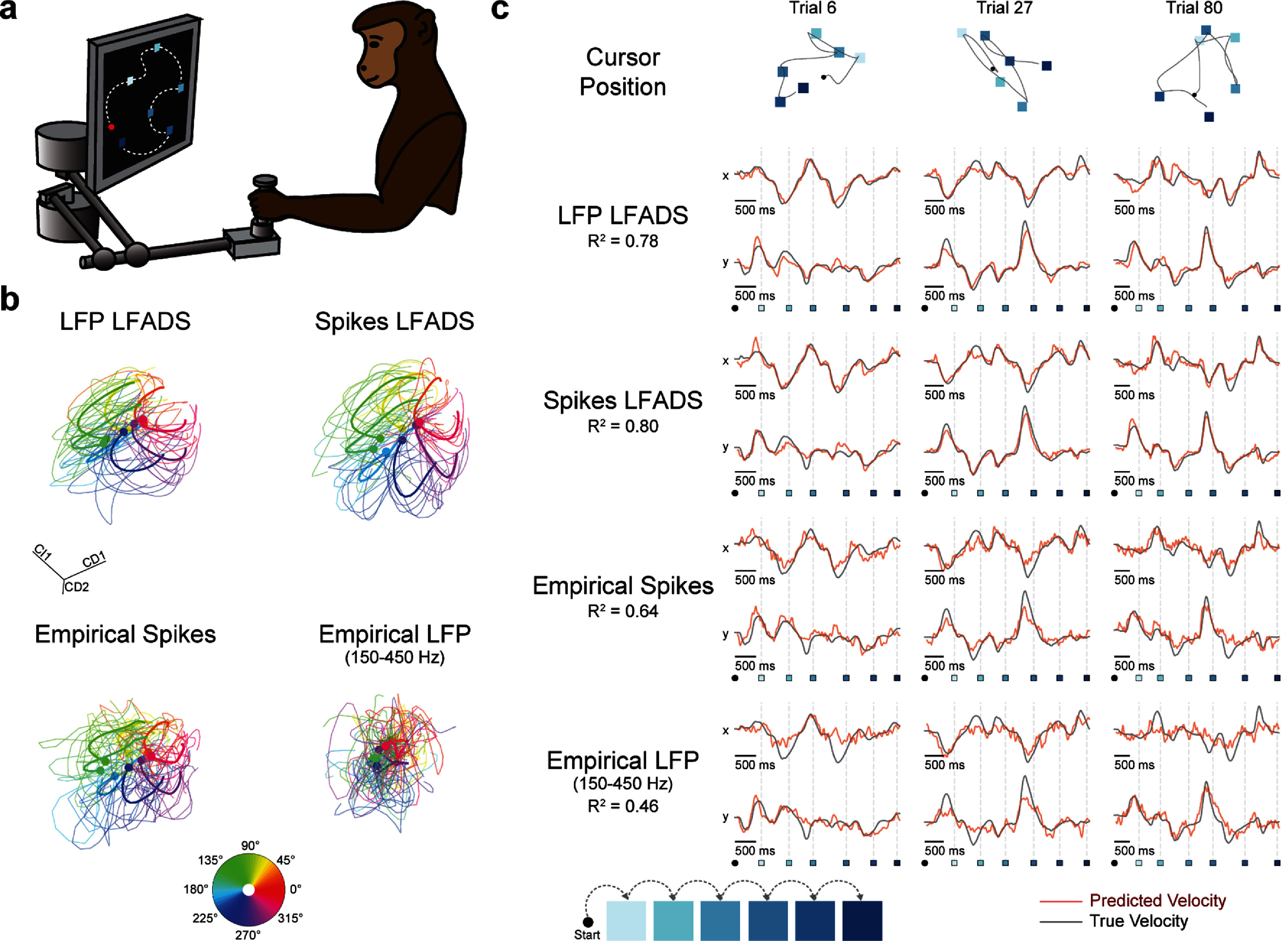
LFP-based dynamics models uncover neural dynamics and accurately decode reach velocity in a monkey random target reaching task. (a) Schematic of random target reaching task. (b) Projections of neural activity onto the top condition-independent dPC and top 2 condition-dependent dPCs. The dPC parameters were determined using the LFP LFADS denoised firing rates and applied to all four representations of neural activity: LFP LFADS rates, Spikes LFADS rates, Empirical Spikes, and Empirical LFP. All neural signals were smoothed with a 30 ms Gaussian kernel prior to PCA for visualization. Each reach is considered the submovement from one target to the next and is aligned in the window 200 ms before and 500 ms after the alignment point. Trajectories are colored by relative angle between the targets. (c) Two-dimensional true cursor position trajectories for three example trials, with targets indicated as blue squares; earlier targets are shaded lighter and later targets are shaded darker (top). Example decoded cursor velocities for three trials of 6 submovements each (bottom). Decoded velocity components are shown for decoders trained to predict from LFP LFADS rates, Spikes LFADS rates, Empirical Spikes smoothed with a 30 ms Gaussian, and Empirical LFP power in the 150–450 Hz frequency band smoothed with a 30 ms Gaussian. The start of each reach is indicated with the round marker, and target acquisition time is shown with a blue-shaded square. True cursor velocity is shown by the black trace, and predicted cursor velocity from each neural signal modality is shown by the red trace.

Next, we wanted to ensure that our conclusions would transfer to a less structured behavior, so we tested LFP-based dynamics models (150–450 Hz) on a monkey random target reaching task (figure 3(a)). On each trial, a monkey controlled a manipulandum to reach six successive targets that appeared one after the other randomly on the screen.

We again aimed to assess how well the models captured the neural data. Because this task was unstructured and not amenable to trial averaging, we chose to summarize the estimated firing rates using demixed principal components analysis (dPCA) [64]. We fit the parameters of dPCA using the firing rates of the LFP LFADS model by binning trials into groups based on relative angle, and then applied those parameters to the Spikes LFADS model rates as well as the Empirical Spikes and Empirical LFP power. We visualized each reach segment between each pair of targets separately, colored by the relative angle between the two targets (figure 3(b)). The LFP LFADS and Spikes LFADS trajectories both captured clear structure consistent with previous analyses of similar tasks [41]. Additionally, both models revealed more obvious task-based organization in the neural data than either the Empirical Spikes or Empirical LFP, further highlighting the benefit of dynamics modeling.

We finally evaluated velocity decoding by training a Wiener filter from each neural signal modality to predict cursor velocity within each trial (figure 3(c)). Predictions from LFP LFADS rates (*R*^2^ = 0.78) were comparable in accuracy and structure to those from Spikes LFADS (*R*^2^ = 0.80). They again exceeded that of both the Empirical Spikes (*R*^2^ = 0.64) and the Empirical LFP power (*R*^2^ = 0.46).

### 3.4. LFP-based dynamics models achieve high-performance reconstruction and accurate decoding of human attempted speech

**Figure 4.**
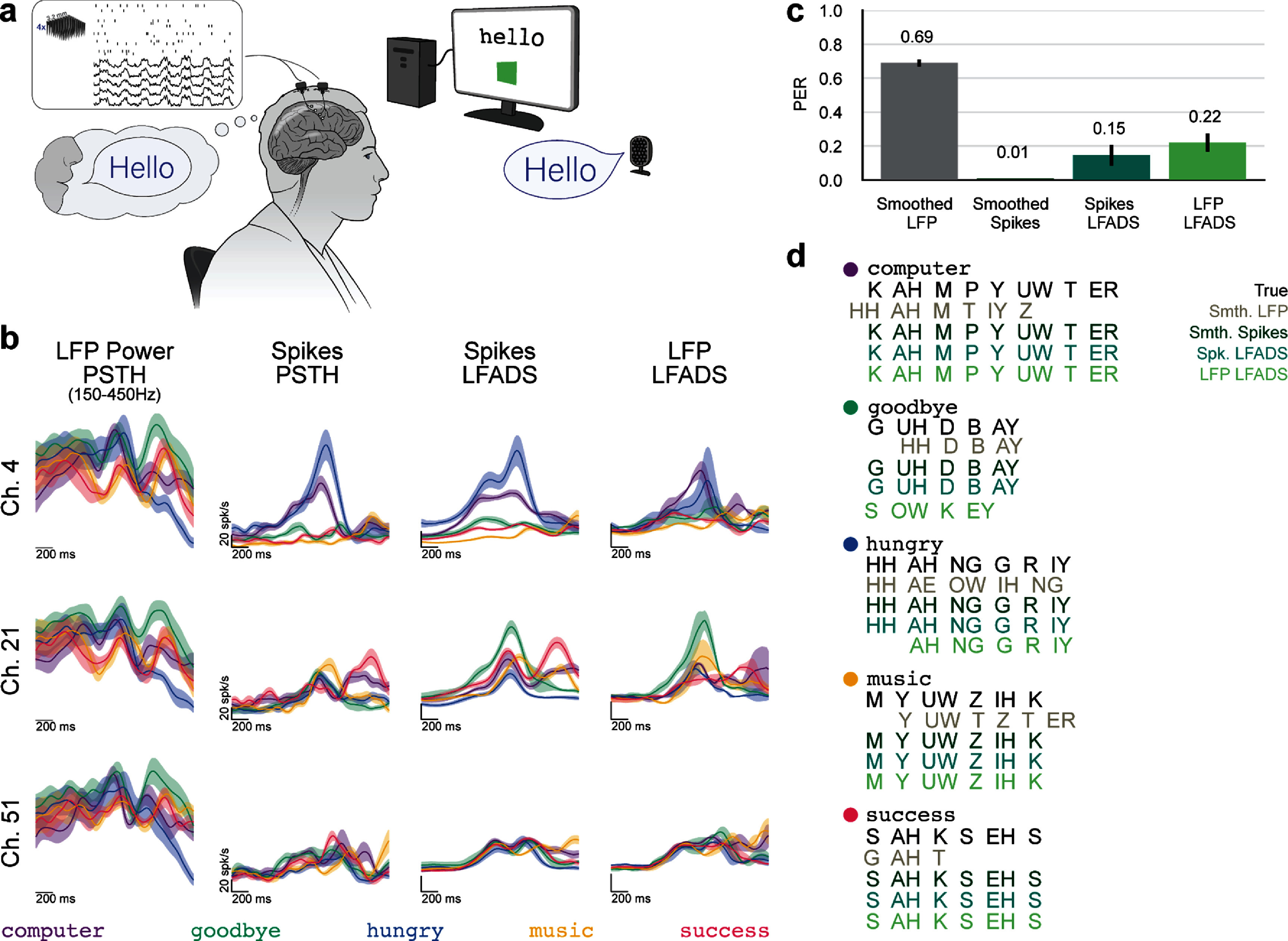
LFP-based dynamics models reconstruct firing rates and enable phoneme decoding in an attempted speech task. (a) Schematic of a trial in the attempted speech task, in which participant T16 was asked to attempt to say one of fifty words. (b) Example PSTHs for three example channels from a single session for smoothed LFP power, smoothed spikes, Spikes LFADS rates, and LFP LFADS rates trained using LFP power from 150 to 450 Hz. Each color is a different word (see legend at bottom), with solid lines indicating the trial average of neural activity for a given word 1000 ms before to 1000 ms after the computed speech onset time and shaded regions representing the standard error of the mean. (c) Validation phoneme error rate (PER) for models trained with each of the four input features: Smoothed LFP power, Smoothed Spikes, Spikes LFADS rates, or LFP LFADS rates. Five decoders were trained with different random seeds. The height of each bar indicates the mean PER across the five decoders and the black error bar indicates one standard deviation. (d) Example decoded outputs for each of the four input signal types in ARPAbet notation [62].

Finally, we aimed to assess the utility of LFP-based dynamics models (150–450 Hz) in a more complex speech task performed by a human participant (figure 4(a)). We performed an offline analysis of an open-loop attempted speech task in which participant T16 was asked to attempt to say one word from a 50-word vocabulary [14] on each trial. In a closed-loop version of the task, the only difference is that decoded phonemes are displayed to the participant following vocalization. The participant does not receive decoder feedback during vocalization in either version of the task, which yields a close correspondence between neural activity in the open- and closed-loop tasks and suggests that the offline decoding results, which are by nature open-loop, have a high likelihood of translating to online (closed-loop) performance.

We again visualized the consistency of PSTHs for the empirical and LFADS output signals (figure 4(b)). We found that LFP power appeared quite consistent across channels, and that there was a greater structural difference between the LFP Power PSTHs (the input signal) and the Spikes PSTHs (the output signal) than in the reaching datasets. Despite these features, there remained a strong qualitative similarity between the LFP LFADS and Spikes LFADS PSTHs.

We next trained the phoneme decoder to predict the intended sequence of phonemes from each of the four neural signal modalities. Our dataset consisted of 400 total trials (8 repeats of each word), of which we reserved 20% for decoder validation. We trained decoders with five different random seeds to ensure consistency of the reported prediction accuracy on validation trials as phoneme error rate (PER) (figure 4(c)). We found that Smoothed LFP yielded the lowest performance (highest error rate) of all four possible signals (mean PER = 0.69 ± 0.02). Smoothed Spikes consistently yielded the highest performance (lowest error rate; mean PER = 0.01 ± 0).

LFP LFADS rates (mean PER = 0.22 ± 0.05) and Spikes LFADS rates (mean PER = 0.15 ± 0.06) offered similar levels of error (p = 0.06 in two-sided Wilcoxon signed-rank test). The consistency in performance between Spikes LFADS and LFP LFADS indicates that the two model types produced outputs that encode similar amounts of phoneme-related information (example phoneme predictions shown in figure 4(d)). The increase in PER from smoothed spikes to both types of LFADS model outputs indicates that the dynamical models may fail to capture some information that is important for phoneme decoding, and that further innovations in dynamics models or adjustments to phoneme decoders are needed.

### 3.5. Discussion

We introduced a new paradigm for training LFP-based dynamics models to reconstruct spiking activity with the goal of reducing power consumption while maintaining offline decoding performance with respect to spikes-based models. In our tests, LFP-based dynamics models performed comparably to spikes-based dynamics models and dramatically better than LFP power alone for tasks encompassing nonhuman primate reaching and human speech. Importantly, this performance can be maintained by running model inference with signals acquired with much lower power than those used with traditional spikes-based decoders.

While in this work we perform experiments using LFADS as our dynamics model, these results may hold with other base neural dynamics models such as dynamics models based on neural ordinary differential equations [44, 45] or feedback control algorithms [42], or with transformer models that use large context windows to denoise neural data like the Neural Data Transformer (NDT) [47]. Such models may provide additional or alternative benefits when compared to LFADS, such as interpretability, inference speed, or flexible sequence lengths. In addition, our demonstration that a continuous-valued signal can be used to reconstruct spikes may prompt further studies of the utility of using smoothed spikes, normalized spikes, or SBP as input signals to neural population dynamics models.

Neural dynamics models can also achieve spatio-temporal super-resolution by inferring missing samples from high-channel-count time series datasets [65]. Such an approach may be advantageous in increasing the number of channels that can be used for decoding while keeping power consumption constant: each channel could be sparsely sampled in time so long as neural dynamics models are trained to infer the missing timesteps. In combination with our efforts to plausibly lower wireless iBCI recording device power consumption, super-resolution training approaches may be particularly useful for decoding applications where channel count has been shown to be an important factor in performance, such as in speech decoding [19].

A recent scientific study investigated the relationship between various frequency bands of LFPs and spiking activity by computing the correlation between LFP power and the principal components of spiking activity [25, 66]. Despite the difference between this technique and our approach, which uses LFP power to nonlinearly reconstruct firing rates and subsequently evaluates behavioral decoding accuracy, we find a remarkably similar relationship between motor cortical LFPs and spiking activity. Both the previous and current study find that higher frequency bands have the strongest relationship, middle frequency bands have a near-zero relationship, and very low frequency bands have a moderate relationship. Pushing forward a rich existing body of work [27, 28, 30–33], future studies may further illuminate the link between these frequency bands of LFPs, spikes, and behavior.

A commonly cited advantage of LFPs is their robustness over time. While spike detection is sensitive to recording interface instabilities due to microshifts in array position or changes in array or tissue properties over time [67, 68], LFPs tend to exhibit both higher longevity, as they can be recorded more reliably despite changes in array properties, and stability, as they can be decoded with more consistent performance over time [36–38]. As a result, our LFP-based dynamics modeling approach may have further benefits for stable iBCI decoding, both on its own and in combination with manifold alignment approaches [56, 69, 70]. In addition, the robust qualities of the LFP signal may allow for unsupervised aggregation of data across sessions, in comparison to approaches like LFADS ‘stitching’ that have previously required knowledge of task structure [40]. LFP-based dynamics models may also leverage LFP longevity—for example, by using a model trained on data from early in the device lifetime, our approach may sustain decoding performance after array degradation has occurred such that spikes can no longer reliably be detected, extending upon previous spikes-based approaches [71].

Recent works have also suggested that neural activity patterns underlying similar behaviors may be shared to some extent across individuals [72, 73]. This suggests that dynamics models, including our LFP-based formulation, may transfer across individuals; that is, if a model is trained using the LFPs and spikes from one individual, it may be possible to reconstruct neural firing rates from the LFPs of another individual. If this is the case, long-term investigations may explore whether it is possible to estimate spikes from other sources of LFPs with different properties than the signals used here, such as that recorded using electrocorticography, as signals recorded from these devices typically cannot yield high-fidelity spiking activity on their own.

Our offline measures of the performance of LFP-based dynamics models demonstrate their potential to combine the power benefits of LFPs with the decoding benefits of spiking activity. Future investigation into the benefits of these models during online iBCI use will help us understand how they may influence wireless device development and whether they may enable better real-world devices.

## Figures and Tables

**Table:** 

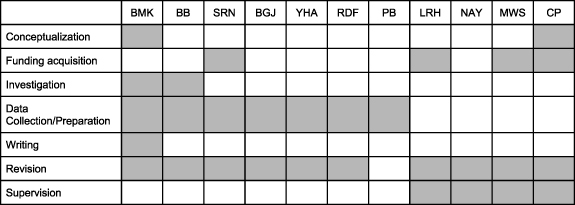

## Data Availability

The data that support the findings of this study are openly available at the following URL/DOI: https://zenodo.org/records/11550255 and https://zenodo.org/records/11397392.

## Code availability

LFADS code with the described functionality is available at: https://github.com/arsedler9/lfads-torch.

Code for reproducing the power calculations in figures 1(b) and (c) is available at: https://github.com/snel-repo/power-calculations/tree/main.
